# Endothelial Progenitor Cells Do Not Originate From the Bone Marrow

**DOI:** 10.1161/CIRCULATIONAHA.119.042351

**Published:** 2019-10-28

**Authors:** Takeshi Fujisawa, Olga Tura-Ceide, Amanda Hunter, Andrew Mitchell, Alex Vesey, Claire Medine, Susan Gallogly, Patrick W.F. Hadoke, Charlotte Keith, Anne Sproul, Huw Roddie, Grant McQuaker, Ian Wilmut, Nicholas L. Mills, Mairi Brittan

**Affiliations:** 1 British Heart Foundation Centre for Cardiovascular Science (T.F., A.H., A.M., A.V., C.M., S.G., P.W.F.H., N.L.M., M.B.), University of Edinburgh, United Kingdom.; 2 MRC Centre for Regenerative Medicine, Scottish Centre for Regenerative Medicine (I.W.), University of Edinburgh, United Kingdom.; 3 Usher Institute for Population Health Sciences & Informatics (N.L.M.), University of Edinburgh, United Kingdom.; 4 Department of Pulmonary Medicine, Hospital Clinic-Institut d’Investigacions Biomèdiques August Pi i Sunyer (IDIBAPS), University of Barcelona, Barcelona and Centro de Investigación Biomédica en Red de Enfermedades Respiratorias (CIBERES), Madrid, Spain (O.T.-C.).; 5 South East Scotland Cytogenetics Service (C.K.), Western General Hospital, Edinburgh, United Kingdom.; 6 Department of Haematology (A.S., H.R.), Western General Hospital, Edinburgh, United Kingdom.; 7 Bone Marrow Transplant Unit, Beatson West of Scotland Cancer Centre, Glasgow, United Kingdom (G.M.).

**Keywords:** bone marrow cells, cell- and tissue-based therapy, endothelial progenitor cells

Endothelial progenitor cells (EPCs) are thought to originate from the bone marrow, mobilize in response to ischemia, and home to sites of vascular injury. Despite uncertainty regarding their origin, phenotype, and therapeutic viability, there remains great interest in harnessing EPCs to promote vascular regeneration. Autologous bone marrow cells have been delivered to thousands of patients, on the premise that these populations contain functional EPCs, with conflicting results.^1^ One potential explanation for the lack of consistent benefit is that bone marrow is not the origin of circulating EPCs. Indeed, although late-outgrowth endothelial cells can be readily isolated from cord and peripheral blood,^2,3^ we have not been able to obtain endothelial cells from the culture of bone marrow.^3^ These findings suggest that circulating EPCs arise from an alternative niche in the vessel wall.

To define EPC origin, we recruited 5 male participants (46±7 years) who had undergone allogeneic bone marrow transplant from female donors for the treatment of hematological malignancy 12 to 120 months previously. The study was performed with approval from our research ethics committee and with written informed consent. Complete donor chimerism was demonstrated in all participants at the time of enrollment. Early- and late-outgrowth endothelial cells were isolated from whole blood,^2,3^ and vessel wall endothelial cells were harvested from forearm veins using a J-shaped guidewire and expanded in culture. The contribution of bone marrow cells to each lineage was assessed by using fluorescence in situ hybridization to detect the X and Y chromosomes, and supported by live cell imaging, flow cytometry, and immunofluorescence staining. Genotype was further analyzed by short tandem repeat analysis using multiplex polymerase chain reaction amplification and detection of DNA sequences of loci that frequently contain polymorphisms. Clonogenic potential at the single-cell level was quantified in each lineage, and the origin of clonogenic progenitors was assessed by fluorescence in situ hybridization.

All early-outgrowth cells had an XX genotype consistent with bone marrow origin, formed clusters of spindle-shaped cells expressing high levels of the pan-leukocyte antigen CD45 rather than endothelial antigens, and did not undergo proliferation or clonogenic expansion (Figure, A and B). Therefore, early-outgrowth cells, previously described as endothelial cell colony-forming units, are hematopoietic and not the progeny of circulating EPCs. In contrast, all vessel wall endothelial cells had an XY genotype, confirming that they were not derived from bone marrow. These cells proliferated in culture to form a cobblestone monolayer with ubiquitous expression of CD31. During early passages, late-outgrowth endothelial cells had a mixed genotype with both XX and XY cells, although the proportion of cells with an XX genotype decreased from 24.8±4.4% to 0.8±0.5% by the third passage (*P*<0.01) (Figure, B). It is important to note that, of those expressing CD31, 99.3±0.7% had an XY genotype at the third passage and therefore did not arise from bone marrow (Figure, C). In contrast, those that did not express CD31 had an XX genotype and were likely contaminating hematopoietic cells commonly found by using this isolation protocol (Figure, D).^2^ These cells expressed CD45, were observed overlying the endothelial monolayer in 3-dimensional confocal z-stacks (Figure, E), and were diminished from passage 1 to 3 (16.5±9.1% versus 1.8±0.9%; *P*<0.05), presumably because of their lack of proliferative capacity and inability to survive in endothelial-specific growth conditions.

**Figure.**
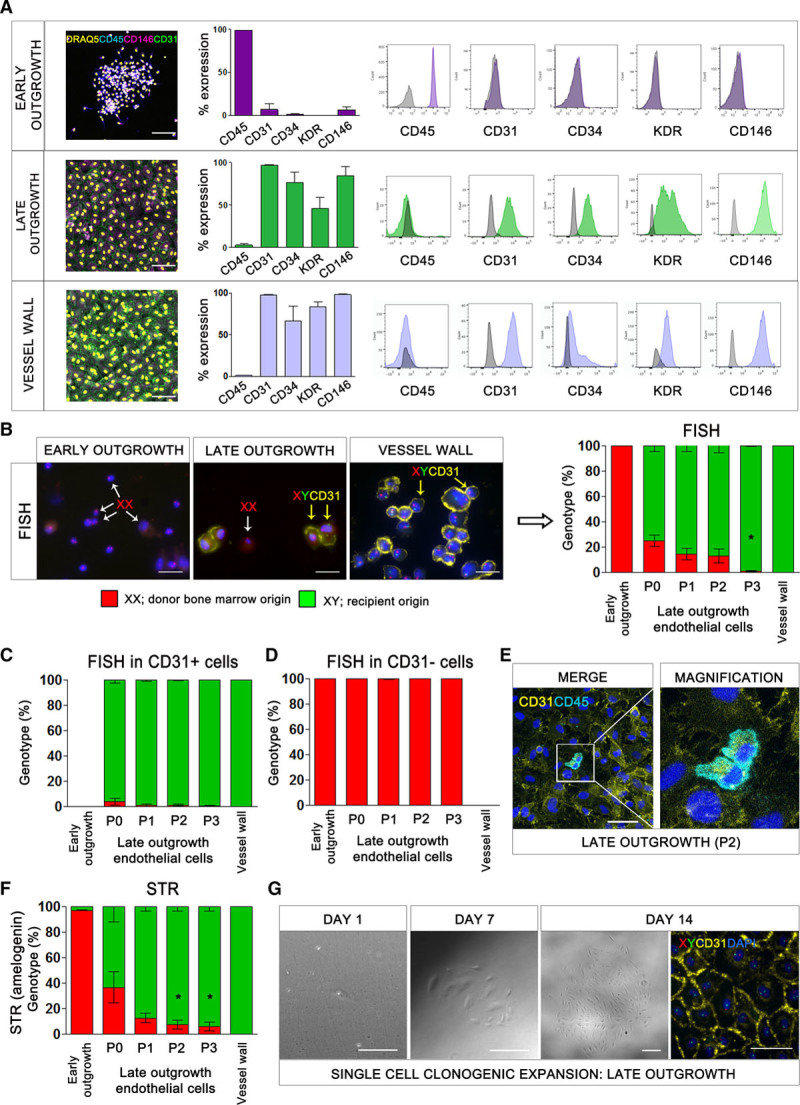
**The origin of endothelial progenitor cells.** **A**, Flow cytometric analysis of early-outgrowth cells, late-outgrowth endothelial cells, and vessel wall endothelial cells with antibodies to CD45, CD31, CD34, KDR, and CD146. Immunofluorescence staining for viable nuclei (DRAQ5, yellow), CD45 (blue), CD146 (magenta), and CD31 (green). Scale bar: 500 µm. **B**, Fluorescence in situ hybridization (FISH) for the X and Y chromosomes combined with CD31 staining of early-outgrowth cells (day 5), late-outgrowth endothelial cells (passage 1), and vessel wall endothelial cells (passage 1) in male patients with sex-mismatched bone marrow transplants. Representative images show Y chromosome (green), X chromosome (red), CD31 (yellow), and nuclei (DAPI, blue). Examples of CD31-negative cells with an XX genotype (white arrows) and CD31-positive cells with an XY genotype (yellow arrows) are shown. Scale bar: 20 μm. *P<0.001, one-way ANOVA. Genotype of early-outgrowth cells, late-outgrowth endothelial cells, and vessel wall endothelial cells with (**C**) and without (**D**) CD31 expression. **E**, Immunofluorescence for CD45 (blue) and CD31 (yellow) in late-outgrowth endothelial cells. Scale bar: 50 μm. **F**, Short tandem repeat analysis for the sex-specific locus, amelogenin. The proportion of cells with XX and XY genotype was calculated from polymerase chain reaction products of 104 and 110 base pairs corresponding to the X and Y chromosomes, respectively. *P<0.05, one-way ANOVA. **G**, Images showing expansion of a colony of late-outgrowth endothelial cells from a single endothelial progenitor cell of recipient origin (XY genotype). Y chromosome (green), X chromosome (red), CD31 (yellow), and nuclei (DAPI, blue). Scale bar: 100 μm. DAPI indicates 4′,6-diamidino-2-phenylindole; and STR, short tandem repeat.

Short tandem repeat analysis of the sex-specific amelogenin gene locus was consistent with fluorescence in situ hybridization (Figure, F), confirming the XX genotype of early-outgrowth cells, the XY genotype of vessel wall endothelial cells, and that late-outgrowth endothelial cells were initially of mixed genotype with a declining fraction of contaminating XX cells between passages 1 and 3 (36.3±2.2% to 5.9±3.6%, *P*<0.05). Clonogenic colonies expanded from single cells were only obtained for late-outgrowth endothelial cells (4/5 participants, 8.8±4.0% efficiency) (Figure, G). All clones expressed CD31 and were entirely XY in genotype.

Although our study includes a small number of participants, we have systematically studied the origin of EPCs in people with sex-mismatched bone marrow transplantation by using 2 distinct but complementary methods. Although endothelial cells can be obtained from a circulating progenitor and are capable of clonal expansion, these cells do not share the genotype of the transplanted bone marrow. We conclude that EPCs in circulation do not originate from the bone marrow.

Our findings contrast those of Lin and colleagues.^4^ They recognized that single-cell culture would be necessary to definitively address whether endothelial cells capable of clonal expansion derive from bone marrow. These methods were used in our analysis demonstrating that all clones formed from single cells were derived from the recipient rather than from donor bone marrow. Our findings were internally consistent and clear, and in agreement with recent evidence showing that endogenous neovascularization in the heart is driven by tissue-resident EPCs without a direct contribution from bone marrow cells.^5^ This represents a paradigm shift that requires a reevaluation of our approach to harness EPCs for therapeutic vascular regeneration.

## Acknowledgments

The authors acknowledge the imaging facility and the flow cytometry facility at the MRC Centre for Regenerative Medicine at the University of Edinburgh for technical support. Prof Kuramoto at Kitasato University in Japan is gratefully acknowledged for support to Dr Fujisawa.

## Sources of Funding

This research was supported by the Chief Scientist Office (CZB/4/812), and the British Heart Foundation through Intermediate (FS/16/4/31831) and Senior (FS/16/14/32023) Research Fellowships and through a Cardiovascular Regenerative Medicine Centre Award (RE/18/5/34216) and Research Excellence Award (RE/18/5/34216).

## Disclosures

None.
